# Normalization methods in time series of platelet function assays: A SQUIRE compliant study

**DOI:** 10.1097/MD.0000000000004188

**Published:** 2016-07-18

**Authors:** Sven Van Poucke, Zhongheng Zhang, Mark Roest, Milan Vukicevic, Maud Beran, Bart Lauwereins, Ming-Hua Zheng, Yvonne Henskens, Marcus Lancé, Abraham Marcus

**Affiliations:** a Department of Anesthesiology, Intensive Care, Emergency Medicine and Pain Therapy, Ziekenhuis Oost-Limburg, Genk, Belgium; b Department of Critical Care Medicine, Jinhua Hospital of Zhejiang University, Zhejiang, P.R. China; c Synapse Research Institute, Maastricht, The Netherlands; d Department of Organizational Sciences, University of Belgrade, Belgrade, Serbia; e Department of Infection and Liver Diseases, Liver Research Center, Wenzhou Medical University, Wenzhou, China; f Central Diagnostic Laboratory, Maastricht University Medical Centre (MUMC+); g Department of Anaesthesiology & Pain Treatment, Maastricht University Medical Centre, Maastricht, The Netherlands; h Department of Anesthesiology, ICU and Perioperative Medicine, HMC, Doha, Qatar.

**Keywords:** aggregometry, data space, high-dimensional, multivariate, normalization, platelets, thromboelastometry

## Abstract

Platelet function can be quantitatively assessed by specific assays such as light-transmission aggregometry, multiple-electrode aggregometry measuring the response to adenosine diphosphate (ADP), arachidonic acid, collagen, and thrombin-receptor activating peptide and viscoelastic tests such as rotational thromboelastometry (ROTEM).

The task of extracting meaningful statistical and clinical information from high-dimensional data spaces in temporal multivariate clinical data represented in multivariate time series is complex. Building insightful visualizations for multivariate time series demands adequate usage of normalization techniques.

In this article, various methods for data normalization (z-transformation, range transformation, proportion transformation, and interquartile range) are presented and visualized discussing the most suited approach for platelet function data series.

Normalization was calculated per assay (test) for all time points and per time point for all tests.

Interquartile range, range transformation, and z-transformation demonstrated the correlation as calculated by the Spearman correlation test, when normalized per assay (test) for all time points. When normalizing per time point for all tests, no correlation could be abstracted from the charts as was the case when using all data as 1 dataset for normalization.

## 1 Introduction

Alterations in platelet function are a common finding in surgical procedures involving cardiopulmonary bypass (CPB). Routine laboratory assays such as activated clotting time, activated partial thromboplastin time, prothrombin time, or platelet count do not provide sufficient specificity and/or sensitivity to assess coagulation and platelet disorders related to the surgical intervention. Platelet function can be quantitatively assessed by more specific assays such as light-transmission aggregometry, multiple-electrode aggregometry measuring the response to ADP, arachidonic acid, collagen, and thrombin-receptor activating peptide and viscoelastic tests such as rotational thromboelastometry (ROTEM). With more data involved, the task of extracting meaningful statistical and clinical information from high-dimensional data spaces, wherein each patient at a certain point in time is defined by hundreds or thousands of measurements, becomes more complex. The massive growth of data set size in health care, in number of records and attributes, has triggered the development of various Big Data platforms that employ parallel data analytics algorithm with a high potential for revealing meaningful clinical information through pattern discovery.^[1]^ Similarly, a high number of attributes triggers the “curse of dimensionality” phenomenon that often prevents building of predictive models and meaningful patterns with good generalization performance (predictions on new data). As such, the use of data dimensionality reduction procedures becomes a necessity for employment of Big Data analytics in medical practice.^[2]^ Selection of important attributes and samples is especially difficult in temporal multivariate clinical data represented in multivariate time series. This task, most often cannot be adequately addressed only by data driven methods and demands deep understanding and involvement of clinical knowledge. Because of that it is of critical importance to provide comprehensible and robust visualizations of the insights obtained by data driven methods, so they could be complemented with clinical knowledge and serve as in decision support. Building insightful visualizations for multivariate time series demands adequate usage of normalization techniques, because different natural scales of original attributes can hide important correlations or temporal trends.^[3]^ However, in current state-of-the-art scientific output, researchers often do not address this problem (adequate normalization of data at hand) in a rigorous manner, potentially leading to biased conclusions.

Optimal implementation of current and new hemostasis and coagulation assays requires quantitative analysis of the involvement of all useful attributes. In this article, several methods for data normalization are presented discussing the most suited approach for platelet function data series.^[4]^ Additionally, a visualization was created, enabling examining multivariate patient data over time more accurately and efficiently than current tabular visualizations.^[5]^

## 2 Materials and methods

### 2.1 Study design and patients

From January 2013 until January 2014, a single-centre, longitudinal observational study collected data from 20 patients at the Maastricht University Medical Centre, after approval by the local medical ethics committee (NTR 4238). Adult patients scheduled for elective CABG with CPB, and a preoperative PlatCt (platelet count) of ≥250 × 10^9^/L were included. Exclusion criteria consisted of emergency surgery, chronic thienopyridine APT, not discontinued at least 5 days prior to surgery, the use of any other anticoagulation drug other than prophylactic low-molecular-weight heparins, congenital disorders of the haemostatic system, and detection of an infection prior to surgery. Eligible patients were recruited on the medical ward the day prior to surgery where they provided written informed consent. Data collection was covered for 3 consecutive days, starting on the day prior to surgery until 24 hours postoperatively. The dataset used in this article consisted of 20 patients and 171 attributes from 3 time points: S1 (before surgical incision), S2 (after weaning from CPB), and S3 (24 hours postoperative).

### 2.2 Blood collection and laboratory analyses

Blood samples were collected in vacuum tubes, using a VenoJect Quick Fit luer adapter (XX-MN2000Q, Terumo Medical, Leuven, Belgium). Following discarding 10 mL of blood at each time point, 4 mL whole blood was collected in a K2EDTA 7.2 mg BD Vacutainer (Ref.: 368861, Becton, Dickinson & Company, Plymouth, UK), 4.5 mL whole blood in a sodium citrate 3.2% BD Vacutainer (Ref.: 367714, Becton, Dickinson & Company), and 3 mL whole blood in a hirudin 15 μg/mL Vacutainer (Ref.: MP0600, Verum Diagnostica GmbH, Munich, Germany). Blood samples were directly transported to the laboratory and analyzed within 2 to 4 hours after collection to allow for minimal necessary resting time for PF tests.

#### 2.2.1 Standard hematological analyses

EDTA–anticoagulated blood was used for cytometric analysis using a whole blood counter Sysmex XE 2100 (Sysmex, Kobe, Japan) to obtain a whole blood count.

#### 2.2.2 Light transmission aggregometry (LTA)

Citrate–anticoagulated whole blood was centrifuged 10 minutes at 170*g* to obtain PRP. The remaining blood was centrifuged twice more, 5 minutes at 2.500*g* followed by 10 minutes at 10.000*g*, providing platelet-poor plasma (PPP) as reference material. Platelet aggregation was subsequently measured in test cuvettes (Ref.: HB-5538-FG, Hart Biologicals Ltd, Hartlepool, UK) filled with nonadjusted PRP, using the Platelet Aggregometer PAR-4 (Ref.: 50.000.1070, Hart Biologicals Ltd), after addition of platelet agonists: AA (Ref.: LS101297, Bio/Data Corporation, Horsham, PA), ADP (Ref.: HB-5502-FG, Hart Biologicals Ltd), COL (Ref.: CH 385, CHRONO-LOG Corporation, Havertown, PA) and TRAP (Ref.: H-8105.0001, BACHEM, Bubendorf, Switzerland) in final concentrations of 1 mM AA, 5 μM ADP, 2 μg/mL COL, and 20 μM TRAP, respectively.

#### 2.2.3 Multiple electrode impedance aggregometry (MEIA)

Platelet aggregation was measured by MEIA principle using the Multiplate multiple electrode aggregometer (Roche Diagnostics, Almere, The Netherlands). Similar to LTA measurements, platelet aggregation was analyzed using the following agonists: AA at a final concentration of 0.5 mM (ASPI-Test; Ref.: MP0210, Dynabyte Medical, Munich, Germany), ADP at a final concentration of 6.4 μM (ADP-Test; Ref.: MP0220, Dynabyte Medical), COL at a final concentration of 3.2 μg/mL (Ref.: 385, Chronolog-PAR, Stago BNL, Leiden, The Netherlands) and TRAP at a final concentration of 32 μM (TRAP-Test; Ref.: MP0250, Dynabyte Medical). All samples were measured after a resting period of 30 minutes following blood collection.

#### 2.2.4 Rotational thromboelastometry (ROTEM)

Thrombus formation was measured by ROTEM (Tem International GmbH, München, Germany). Standard assays and reagents (Tem International GmbH) were used according to the manufacturer's recommendations: EXTEM, FIBTEM, and HEPTEM. All samples were measured within 1 hour after blood collection. Furthermore, by means of EXTEM and FIBTEM results, the contribution of platelet count to the thrombus formation was calculated as the PLTEM parameter.

### 2.3 Statistical analysis

Analysis was performed by RapidMiner (7.0, Boston, MA). RapidMiner (previously: Rapid-I, YALE) became popular in recent years and is supported by a large scientific community (http://www.kdnuggets.com/2016/02/rapidminer-leader-2016-gartner-mq-advanced-analytics-platforms.html). RapidMiner provides multiple extensions suited for dimensionality reduction.^[1]^ Furthermore the tool enables seamless parameter optimization, being a necessary step for many cutting edge algorithms (e.g., PCA). Data were displayed by Tableau (9.2, Seattle, WA) (https://www.tableau.com/about/press-releases/2016/gartner-positions-tableau-leader-magic-quadrant-bi-and-analytics-platforms). The dashboard developed for this study is publicly accessible on Tableau Public: https://public.tableau.com/views/LineChart_8/Dashboard1?:embed=y&:display_count=yes&:showTabs=y.

### 2.4 Normalization

Normalization is as preprocessing step used to rescale attribute values to fit in a specific range. In data analysis, normalization is a type of data transformation referring to the replacement of a variable by a function of that variable: for example, replacing a variable x by the square root of x or the logarithm of x. In a stronger sense, a transformation is a replacement that changes the shape of a distribution or relationship.

Normalization of the data is of particular importance when dealing with attributes of different units and scales. In some data mining algorithms like K-NN, the input attributes are expected to be numeric and normalized because the algorithm compares values of different attributes and calculates distance between data points. Data normalization methods enable to bring all of the variables into proportion with one another.^[6]^ Finding an appropriate method to deal with time series normalization is not an easy task because most of the traditional normalization methods make assumptions that do not hold for most time series. The first assumption is that all time series are stationary, that is, their statistical properties, such as mean and standard deviation, do not change over time. The second assumption is that the volatility of the time series is considered uniform.^[7]^

In this study, normalization is calculated by 4 normalization methods. Each method calculated normalization on the complete dataset, by test for the 3 points in time and by time point for all the tests.

Results of this article were based on the following normalization methods:

z_transformation

This is also called statistical normalization. The purpose of statistical normalization is to convert a data into Normal (Gaussian) distribution with mean = 0 and variance = 1. The formula of statistical normalization is Z = (X − u)/s. Attribute values are considered as vector X which are subtracted by the mean of the attribute values, u, and the difference is divided by the standard deviation, resulting in a vector Z with normal distribution (with zero mean and unit variance), also called Standard Normal distribution, N(0,1). However, the range of the standard Normal distribution is not limited to [0,1]. Limiting the range to −3 and +3 captures 99.9% of the data. This scaling method is useful when the data follows normal distribution, if the data do not follow normal distribution the method is less suitable.

Proportion_transformation

Each attribute value is normalized as proportion of the total sum of the respective attribute, that is, each attribute value is divided by the total sum of the attribute values.

Range_transformation

Range transformation normalizes all attribute values in the user specified range [min,max]. Consider the min–max and the decimal scaling methods, for instance. Their applicability depends on the knowledge of the minimum and/or maximum values of a time series, which is not always possible.

Interquartile_range

Since normalization by range_transformation (described above) only takes into account max and min values for each feature, it may be heavily influenced by outliers in the data. Therefore, another criterion—the interquartile range—is commonly used. It is the distance between the 25th and 75th percentiles (Q3 − Q1). The interquartile range is essentially the range of the middle 50% of the data. Because it uses the middle 50%, the interquartile range is not affected by outliers or extreme values.

Normalized values are represented by polynomial trend lines (polynomial trends in Tableau, have model degrees of freedom of 1 plus the degree of the polynomial).

## 3 Results

Guided by the CRoss-Industry Standard Process for Data Mining (CRISP-DM), the initial dataset (20 patients, 154 attributes) was imported in RapidMiner for data preparation.

The variables (attributes) from standard hematological testing, rotational thromboelastometry, light transmission aggregometry, and multiple electrode aggregometry (units) are: *Laboratory tests*: Hb (mmol/L); Hct (%); PlatCt (×10^9^/L); MPV (fL) *ROTEM (Rotational Thromboelastometry)*: A5 EXTEM (mm); A5 EXTEMtPA (mm); A5 FIBTEM (mm); A5 HEPTEM (mm); A5 PLTEM (mm); A10 EXTEM (mm); A10 EXTEMtPA (mm); A10 FIBTEM (mm); A10 HEPTEM (mm); A10 PLTEM (mm); A20 EXTEM (mm); A20 EXTEMtPA (mm); A20 FIBTEM (mm); A20 HEPTEM (mm); A20 PLTEM (mm); Alpha EXTEM (mm); Alpha EXTEMtPA (mm); Alpha FIBTEM (mm); Alpha HEPTEM (mm); Alpha PLTEM (mm); MCF EXTEM (mm); MCF EXTEMtPA (mm); MCF FIBTEM (mm); MCF HEPTEM (mm); MCF PLTEM (mm); ML EXTEM (%); ML EXTEMtPA (%); ML FIBTEM (%); ML HEPTEM (%); ML PLTEM (%); CT EXTEM (s); CT EXTEMtPA (s); CT FIBTEM (s); CT HEPTEM (s); CT PLTEM (s); CFT EXTEM (s); CFT EXTEMtPA (s); CFT FIBTEM (s); CFT HEPTEM (s); CFT PLTEM (s); *LTA (Light Transmission Aggregometry)*: MA AA (%); MA ADP (%); MA COL (%); MA TRAP (%);*MEA (Multiple Electrode Aggregometry)*: AUC ASP (U); AUC ADP (U); AUC COL (U); AUC TRAP (U) AggrRate AA (*na*); AggrRate ADP (*na*); AggrRate COL (*na*); AggrRate TRAP (*na*).

### 3.1 Correlation

**Figure 1:**
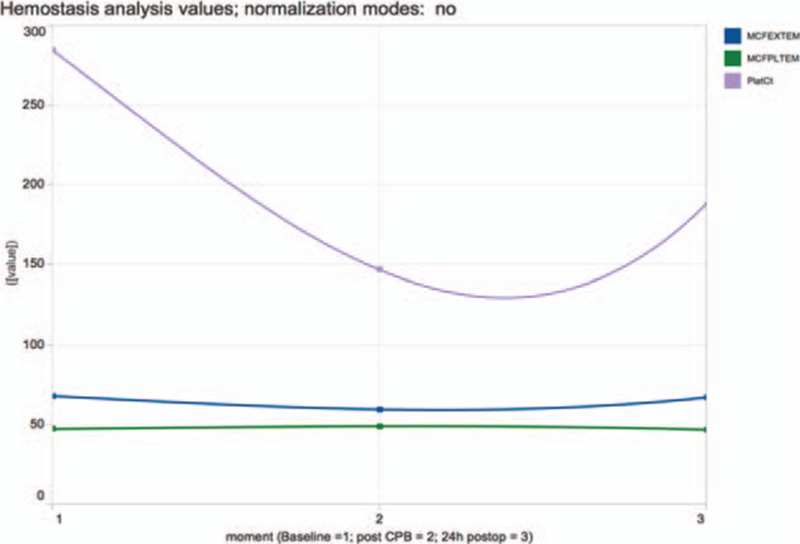
Data of PlatCt, MCF EXTEM, MCF PLTEM for baseline, post-CPB, postop. No normalization. Charts generated by Tableau and available from: https://public.tableau.com/views/LineChart_8/Dashboard1?:embed=y&:display_count=yes&:showTabs=y. Normalization modes: no: without normalization. CBP = cardiopulmonary bypass, MCF = maximum clot firmness, PlatCt = platelet count, postop = postoperative.

Correlations between LTA/MEIA/ROTEM and PlatCt or Hct, were analyzed using Spearman correlation test (Table 1). The Spearman correlation is nonparametric with the exact sampling distribution obtained without requiring knowledge of the joint probability distribution of the parameters. Correlation analysis using Spearman correlation coefficients (Table 1) identified a strong (*r* > 0.40 or *r* < −0.40) correlation between MEIA results and PlatCt for respectively ADP- (*r* = 0.741, *P* < 0.01), COL- (*r* = 0.494, *P* < 0.01), and TRAP-induced (*r* = 0.551, *P* < 0.01) platelet aggregation. With respect to ROTEM measurement, EXTEM MCF (*r* = 0.749, *P* < 0.01) demonstrated a strong correlation with PlatCt (Fig. 1). No moderate (0.30 < *r* < 0.39 or −0.30 > *r* > −0.39) or strong correlations were found between LTA measurement and PlatCt intrinsic to LTA methodology. Further, strong correlation is observed (*r* > 0.40 or *r* < −0.40) between MEIA results and Hct for, respectively, AA- (*r* = 0.492, *P* < 0.01) and ADP-induced (*r* = 0.405, *P* < 0.01) platelet aggregation. Additionally, ROTEM values were correlated with Hct results (FIBTEM MCF *r* = 0.374, *P* < 0.01). Correlation analysis between LTA and Hct was not performed since this assay is performed in PRP.

**Table 1:**
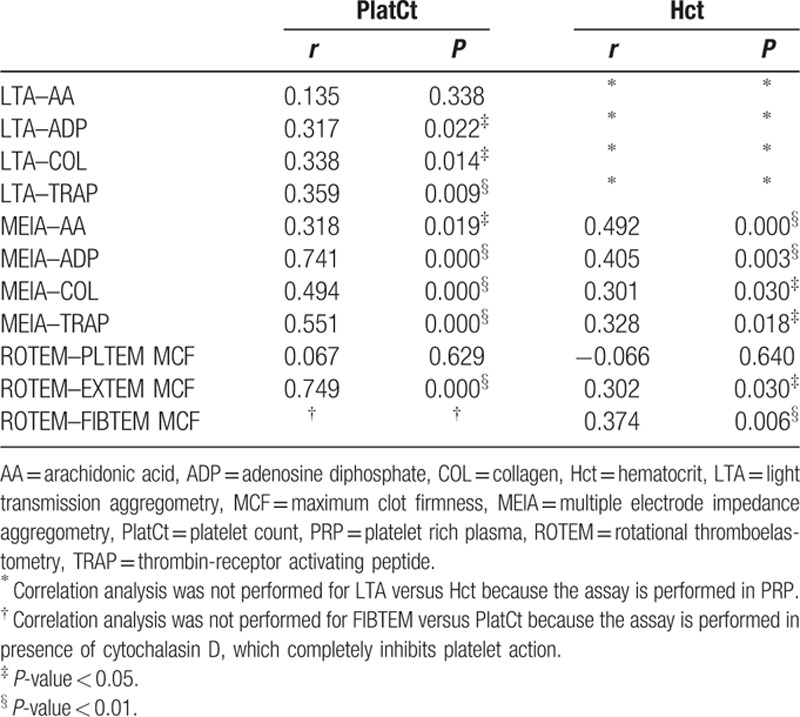
Spearman rank test and *P* values of correlation analyses between LTA, MEIA, and ROTEM versus PlatCt and Hct (n = 20).

In order to illustrate different normalization methods the correlation of PlatCt and ROTEM EXTEM MCF (*r* = 0.749, *P* = 0.000) and ROTEM PLTEM MCF (*r* = 0.067; *P* = 0.629) is studied (Table 1). Each method calculated normalization, on the complete dataset, by test for the 3 points in time and by time point for all the tests. With a strong and significant correlation of PlatCt with ROTEM EXTEM MCF, and a weak, nonsignificant correlation of PlatCt with ROTEM PLTEM MCF, the difference between the 2 features should be clearly to illustrate. With platelet count (PlatCt) expressed as ×10^9^/L (normal range: 150–450) and ROTEM maximal clot formation (MCF) as mm (normal range ROTEM EXTEM MCF: 49–71 and ROTEM PLTEM MCF: 35–45). PLTEM, was calculated by subtracting FIBTEM from EXTEM to distinguish thrombocytopenia from hypofibrinogenemia.^[8]^

**Figure 2:**
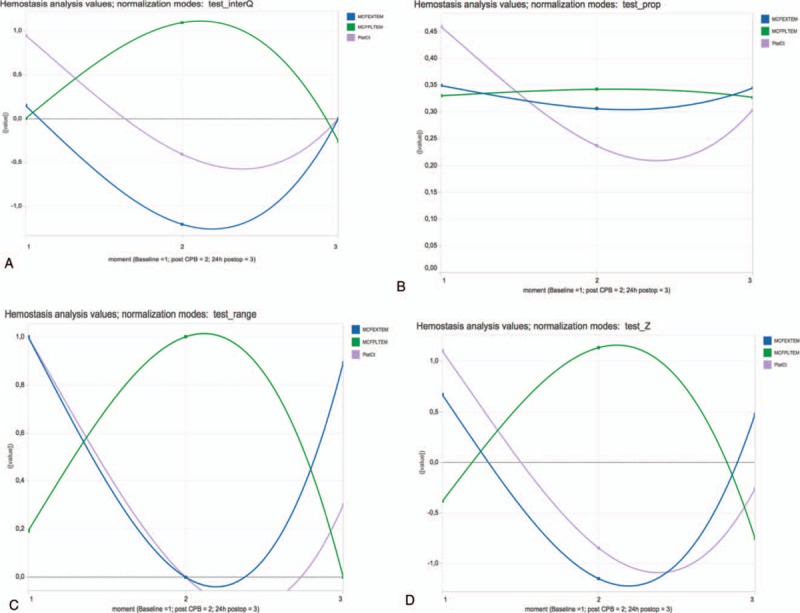
Data of PlatCt, MCF EXTEM, MCF PLTEM for baseline, post-CPB, postop. Normalization by test for the 3 points in time (moments: baseline, post-CPB, postop). Charts generated by Tableau and available from: https://public.tableau.com/views/LineChart_8/Dashboard1?:embed=y&:display_count=yes&:showTabs=y. Normalization mode: test: normalization by test for the 3 points in time (moments: baseline, post-CPB, postop). (A) InterQ: interquartile range; (B) range: range transformation; (C) prop: proportion transformation; (D) Z: z-transformation. CBP = cardiopulmonary bypass, MCF = maximum clot firmness, PlatCt = platelet count, postop = postoperative.

**Figure 3:**
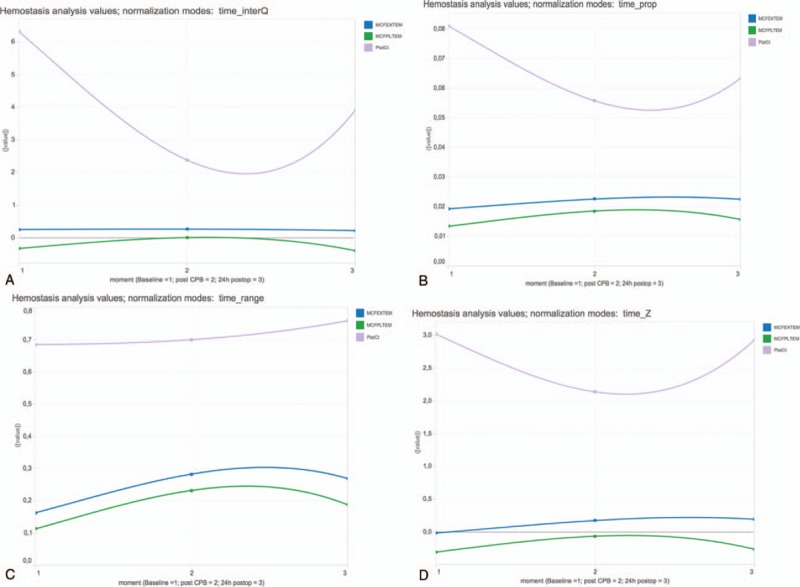
Data of PlatCt, MCF EXTEM, MCF PLTEM for baseline, post-CPB, postop. Normalization by time point for all tests (MCF EXTEM, MCF PLTEM, and PlatCt). Charts generated by Tableau and available from: https://public.tableau.com/views/LineChart_8/Dashboard1?:embed=y&:display_count=yes&:showTabs=y. Normalization mode: time: normalization by time point for all tests (MCF EXTEM, MCF PLTEM, and PlatCt); (A) InterQ: interquartile range; (B) range: range transformation; (C) prop: proportion transformation; (D) Z: z-transformation. CBP = cardiopulmonary bypass, MCF = maximum clot firmness, PlatCt = platelet count, postop = postoperative.

**Figure 4:**
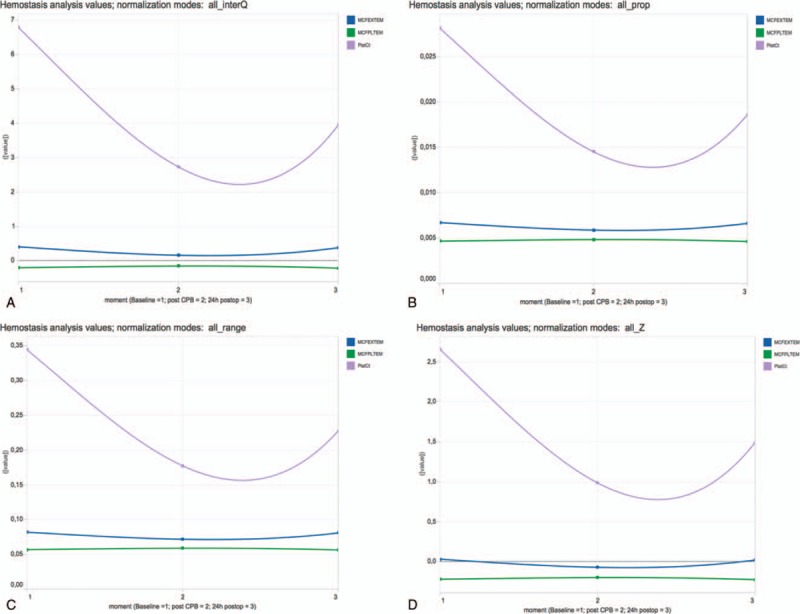
Data of PlatCt, MCF EXTEM, MCF PLTEM for baseline, post-CPB, postop. Normalization on the complete dataset. Charts generated by Tableau and available from: https://public.tableau.com/views/LineChart_8/Dashboard1?:embed=y&:display_count=yes&:showTabs=y. Normalization mode: all: normalization on the complete dataset; (A) InterQ: interquartile range, (B) range: range transformation; (C) prop: proportion transformation; (d) Z: z-transformation. CBP = cardiopulmonary bypass, MCF = maximum clot firmness, PlatCt = platelet count, postop = postoperative.

Polynomial trend lines of PlatCt, ROTEM EXTEM MCF, ROTEM PLTEM MCF values, illustrated without any normalization (Fig. 1), was not able to illustrate correlation of PlatCt and ROTEM EXTEM MCF (Fig. 1). Additionally, differences in unit and scale of PlatCt values compared to ROTEM EXTEM MCF, ROTEM PLTEM MCF values impede a clear display of the time related changes of ROTEM EXTEM MCF, ROTEM PLTEM MCF values (Figs. 2–4).

Similar observations are noticeable, although with different scaling, with normalization of all data by z-transformation (Fig. 4D), range transformation (Fig. 4B), proportion transformation (Fig. 4C), and interquartile range (Fig. 4A).

Analyzing normalization by test for all time points only reveals correlation of PlatCt and ROTEM EXTEM MCF by interquartile range (Fig. 2A), range transformation (Fig. 2B), and z-transformation (Fig. 2D). No correlation is abstracted from visualization by proportion normalization (Fig. 2C).

Analyzing normalization by time point for all tests did not demonstrate correlation of PlatCt and ROTEM EXTEM MCF in any chart (Fig. 3A–D).

## 4 Discussion

In biomedical environments, it is desirable to compare dynamical systems based on their behavior.^[9]^ Similarity of behavior often implies similarity of internal mechanisms or dependency on common extrinsic factors (e.g., LTA and MEIA agonists). Although methods for comparing univariate time series are generally adopted, most dynamical systems in biomedicine are characterized by multivariate time series. Comparison of multivariate time series has been limited to cases where a common dimensionality is shared.^[10]^

Normalization is a generally employed preprocessing technique used to rescale attribute values to fit in a specific range. Normalization of the data is critical when dealing with attributes with different units and scales because certain data mining techniques (e.g., the ones based on distance/similarity calculations) require normalization. However, normalization of biomedical data is often ignored, and this can lead to misinterpretation of the results and ultimately wrong decisions.^[11]^

Finding an appropriate method for time series normalization is not a clear cut task.^[7]^ Most of the traditional normalization methods make assumptions that are lacking in time series. A first assumption is related with the nonstationary property of time series. Stationary processes assume that their statistical properties (e.g., mean and standard deviation), do not fluctuate over time. The second assumption is related with the volatility of the time series which is considered uniform. It is proposed to normalize time series by Adaptive Normalization.^[7]^ In Adaptive Normalization, the original nonstationary time series is transformed into a stationary sequence. This transformation is based on the concepts of moving averages. In this article, moving averages were not implemented because only 3 time points were included. The authors attempted to find the adequate normalization technique(s) illustrating the correlations as calculated for the different features.

When feature values cover a large range, the use of the logarithms of the values rather than the actual values reduces the wide range to a more manageable size. This approach might be suitable for visualization, when using certain analytical methods, normalization becomes essential. Basically, normalization is performed to obtain the same range of values for data mining and machine learning techniques like support vector machine, neural network, etc. This can guarantee stable convergence of weight and biases and speed of the optimization process.

We developed an online dashboard (Tableau) enabling to measure similarity for multivariate time series representations of physiological and laboratory data allowing physicians to identify patients with similar events and/or phenotypes for the purpose of predicting patient outcomes.^[12]^

Polynomial trend lines of PlatCt, ROTEM EXTEM MCF, ROTEM PLTEM MCF values with normalization (by z-transformation, range transformation, proportion transformation, and interquartile range) or without normalization on all values of the dataset, was not able to illustrate correlation of PlatCt and ROTEM EXTEM MCF (Fig. 1). This approach is also not suitable for time series considering the nonstationary property of the data. When normalization was performed for each test separately but for all time points, correlation of PlatCt and ROTEM EXTEM MCF was illustrated by interquartile range (Fig. 2A), range transformation (Fig. 2B) and z-transformation (Fig. 2D). No correlation is abstracted from visualization by proportion normalization (Fig. 2C). Analyzing normalization separately for each time point but for all tests, correlation of PlatCt and ROTEM EXTEM MCF could not be abstracted in any chart (Fig. 3A–D). Besides correlation analysis of features, temporal trend should be considered. The chart illustrating no normalization (Fig. 1) and the chart illustrating normalization performed for each test separately but for all time points (Fig. 2A–D), both ROTEM EXTEM MCF and ROTEM PLTEM MCF have a negative correlation, but on the chart illustrating no normalization (Fig. 1), no clear temporal change of ROTEM EXTEM MCF and ROTEM PLTEM MCF is observed.

## 5 Conclusion

There is no unique assay to quantitatively assess platelet function. Tools as been provided by this study enable clarification of the complex relationship between the various features measured in clinical medicine. Results of multivariate time series are often represented without normalization. Because of different scales of original features, such visualizations most often cannot reveal significant correlations between variables, nor temporal trends. Additionally, many machine learning and data mining algorithms require normalization as preprocessing step in order to provide valid models (i.e., k-means or k-NN) or to allow fast and stable convergence to the optimal solution (i.e., logistic regression). However, there is a multitude of available normalization techniques, and not all of them are suitable for each type of data. In this study, we examined the value of several normalization techniques (z-transformation, range transformation, proportion transformation, and interquartile range) for visualizing correlations and temporal trends of temporal tests. Interquartile range, range transformation, and z-transformation demonstrated correlation when normalized per assay (test) for all time points; when normalizing per time point for all tests, no correlation could be abstracted from the charts as was the case when using all data as 1 dataset for normalization. These conclusions might provide a tool for deeper investigation of potential correlations. Different normalization techniques lead to different views on data.
